# Diabetes mellitus with a duration of 26 years combined with IgA nephropathy: A case report and literature review

**DOI:** 10.3389/fendo.2022.992933

**Published:** 2022-12-23

**Authors:** Yan-Li Gou, Ya-Pu Zhang, Yang Xu, Jian-Dong Li, Yang Huang, Hang Chen

**Affiliations:** ^1^ Department of Nephrology, Affiliated Hospital of Hebei University, Baoding, China; ^2^ Hebei University, Baoding, China

**Keywords:** diabetes mellitus, IgA nephropathy, diabetes mellitus combined with IgA nephropathy, nephrology, case report

## Abstract

**Background:**

The incidence of diabetes mellitus (DM) in China is increasing yearly and has become a major problem plaguing national public health. The diagnosis of diabetic kidney disease (DKD) is based primarily on clinical criteria, and most patients do not receive a formal evaluation by renal biopsy; thus, misdiagnosis and underdiagnosis are common. The incidence of non-diabetic kidney disease (NDKD) is also higher in those with DM. To date, many cases of IgA nephropathy (IgAN) among those with DKD have been reported, while cases of IgAN in patients with long-duration DM who did not develop DKD are less commonly reported.

**Case description:**

A 70-year-old male patient with a diabetes duration of 26 years had proteinuria for one year. The clinical manifestations of nephrotic syndrome and IgAN were confirmed by renal biopsy. The patient received targeted treatment for three years with partial alleviation of proteinuria.

**Conclusion:**

Renal biopsy might aid in the definitive diagnosis of DKD, NDKD, and NDKD combined with DKD. Precise therapy based on renal pathology might help to improve outcomes in the kidney.

## Introduction

Diabetic kidney disease (DKD) has become the most common secondary glomerular disease in elderly patients with kidney disease, and its incidence is increasing year by year (1). The incidence of non-diabetic kidney disease (NDKD) is also higher in patients with type 2 diabetes mellitus (T2DM). According to global reports of renal biopsies and pathological NDKD lesions, the overall prevalence of DKD, NDKD, and DKD combined with NDKD is 41.3%, 40.6%, and 18.1%, respectively. In Asia, Africa (especially Morocco and Tunisia), and Europe, the most common pathological type of isolated NDKD is membranous nephropathy (MN) (2). A Chinese study showed that IgA nephropathy (IgAN) was the most common pathological pattern in young patients with NDRD, being present in 26.3% of such cases (3). NDRD may occur independently in patients who do not have DKD or in combination with DKD. Patients with DKD may or may not also be diagnosed with diabetic retinopathy (DR), and DR may indicate the possibility of DKD; however, the existence of NDRD may not be excluded. Renal biopsy may accurately diagnose DKD, NDKD, and NDKD combined with DKD. For those with NDKD, appropriate treatment may result in improved renal outcomes. The present study reports the clinical diagnosis, treatment process, and outcome of NDKD in one elderly patient with long-duration DM combined with IgAN. In the present patient, accurate diagnosis and treatment based on renal pathology improved the prognosis.

## Case report

**Figure 1:**
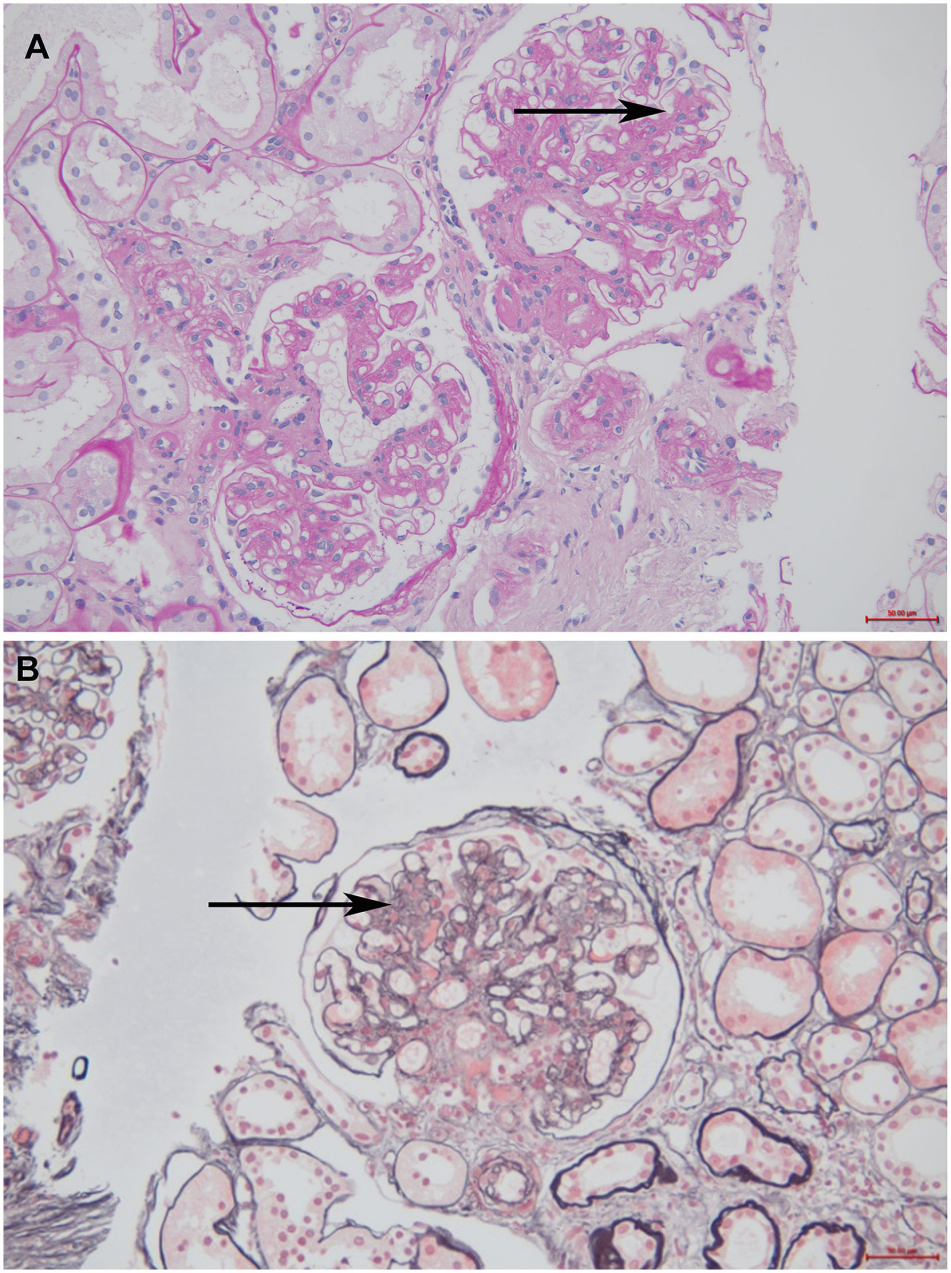
The optical microscope results of the patient. **(A)** Periodic acid-Schiff staining (200× magnification). The black arrow indicates moderate to diffuse proliferation of mesangial cells and stroma **(B)** Periodic acid silver methenamine staining and Masson’s staining (200× magnification). The black arrow indicates severe diffuse proliferation of mesangial cells and stroma.

Patient: Mr. Niu; gender: male; age: 70 years old; occupation: retired medical staff. The chief complaint was intermittent facial and bilateral lower extremity edema. The patient developed facial and bilateral lower limb edema without any known cause one year ago. Increased nocturia was reported, although there was no hematuria, no fever, no malaise, no skin rash, and no photosensitivity. The patient was found to be positive for proteinuria several times. The patient was administered “Bailing Capsules and Shenyankangfu Tablets” orally, but the edema varied between mild and (at times) severe. The outpatient examination results were as follows (2021-10-13): urine protein quantification 5.54 g/24 h/2300 ml, blood glucose (BG) 7.5 mmol/L, glycosylated albumin 21.3%. The patient had a previous history of DM for 26 years with a maximum BG of 16 mmol/L. The patient was treated with Novolin 16 U bid and glargine insulin 12 U qn, together with oral administration of “Metformin”. Fasting BG (FBG) was within the range of 10–15 mmol/L with basically normal postprandial blood glucose (PBG). The patient had a 20-year history of hypertension with maximum blood pressure (BP) of 160/90mmHg. He was medicated with oral administration of Norvasc 5 mg QD and BP was maintained at around 140/80 mmHg. He had a 20-year history of coronary heart disease (CHD) with long-term oral administration of Atorvastatin. A pacemaker had been implanted for eight months due to bradycardia. Patient had undergone cataract surgery in the right eye six months earlier, and suffered from blurred vision in both eyes at time of examination. Personal history: reported no history of smoking and drinking. Marital history: patient married at 29 years of age; spouse had hypertension and DM. Patient had one son, who was healthy. Family history: patient’s father died of unknown cause; mother died of cerebral hemorrhage. Patient’s two brothers died of renal failure and esophageal cancer, respectively. Patient’s two sisters had DM and hypertension respectively before death. Results of physical examination at admission: T 36.5℃, P 66/min, R 18 times/min, BP 153/92 mmHg. The patient was conscious and communicated fluently. The bilateral breath sound was clear without dry and moist rales. The pacemaker rate was 66 times/min. The abdomen was flat, and the liver and spleen were impalpable. Mild pit edema of both lower extremities was observed. Ultrasonography results: normal size of both kidneys, sclerosis plaque formation in the bilateral carotid artery. Chest computed tomography (CT) results: multiple micronodules in both lungs with partial calcification, which were considered to be benign. Post cardiac surgery, aortic and coronary artery sclerosis. No significant changes compared to the examination results on 3 Mar 2021. Electrocardiography (ECG) results: dual-chamber pacing rhythm with normal electrical axis. Routine blood test results: white blood cell (WBC) 5.78×10^9^, red blood cell (RBC) 4.62×10^12^, platelet (PLT) 154×10^9^, Neutrophil (N) 65%. Routine urine test results: urine protein 3+, occult blood, RBC 0.61/Hp, glucose (GLU)2+. Renal function test results: blood urea nitrogen (BUN) 7.63 mmol/L, creatinine (Cr) 57 μmol/L, uric acid (UA) 336 μmol/L. Liver function test results: albumin (ALB) 30 g/L, lipid:total cholesterol (TCH) 5.60 mmol/L, triglyceride (TG) 2.13 mmol/L. The levels of serum albumin, serum creatinine, and urine protein of this patient on different dates are show in **Table 1**. Glycosylated hemoglobin (HBA1c) 7.2%; albumin creatinine ratio (ACR) 5871.94 mg/g. No abnormalities in the following: four items of coagulation, three items of cardiac enzymes, eight items of infection, antineutrophil cytoplasmic antibody (ANCA), anti-phospholipase A2 antibody (PLA2R), antinuclear antibody profile, autoantibodies, blood and urine immunofixation electrophoresis, immunoglobulins, and complements. Impressions at admission: 1. nephrotic syndrome (NS); 2. hypertension, grade 2 with very high risk; 3. DM; 4. CHD; 5. post-pacemaker implantation; 6. post-operation of right cataract surgery. The treatments administered were as follows: 1. monitoring of BP and BG, with a low-salt, low-fat diabetic diet; 2. Atorvastatin for lipid-lowering, Novolin + glargine insulin + Metformin for BG lowering and amlodipine besylate for BP lowering; 3. plan to conduct a renal biopsy to confirm the pathological diagnosis. On 10 Nov 2021, following the completion of relevant examinations, a renal biopsy was conducted. On 12 Nov 2021, the T cell results for tuberculosis were found to be positive, and the department of tuberculosis was consulted. The suggestions were as follows: taking into account the calcific density nodule in the right middle lobe in lung CT and TB-IGRA (+), the status of tuberculosis infection should be considered, and old tuberculosis of the right lung could not be excluded; prophylactic anti-tuberculosis therapy should be conducted through the administration of hormones or immunosuppressants. Results following consultation with the department of ophthalmology were as follows: macular degeneration in both eyes, cataract in the left eye, post-operation of cataract surgery in the right eye. Calcium hydroxybenzene sulfonate was recommended along with regular review. Immunofluorescence (IF): IgA+ IgG± IgM- C3+ C1q- FRA- Alb± κ± λ± IgG_1-_ IgG_2-_ IgG_3-_ IgG_4-_; Light microscopy showed moderate to severe diffuse proliferation of mesangial cells and stroma. The periodic acid-Schiff staining showed moderate to diffuse proliferation of mesangial cells and stroma (**Figure 1A**), and the periodic acid silver methenamine staining and Masson’s staining showed severe diffuse proliferation of mesangial cells and stroma (**Figure 1B**). It was accompanied by diffuse thickening of the basement membrane, small eosinophilic deposits in the subendothelial segment, and microangioma-like expansion of segmental capillary loops. There were vacuoles and granular degeneration of renal tubular epithelial cells, focal atrophy, and a few protein casts were observed in the lumen. Renal interstitial focal lymphocyte and monocyte infiltration with fibrosis, wall thickening of small arteries, intimal fibrosis and sclerosis, and lumen stenosis were also observed.

**Table 1:** The levels of serum albumin, serum creatinine, and urine protein on different dates.

	Feb.02.2021	Jun.16.2021	Nov.10.2021	Dec.17.2021	Jan.14.2022	Mar.01.2022
Albumin (g/L)	36	30	30	31	33	36
Creatinine (μmol/L)	74	65	57	73	81	82
Urine protein (g/24 h)	3.67	6.16	4.12	4.66	2.88	2.6

**Figure 2:**
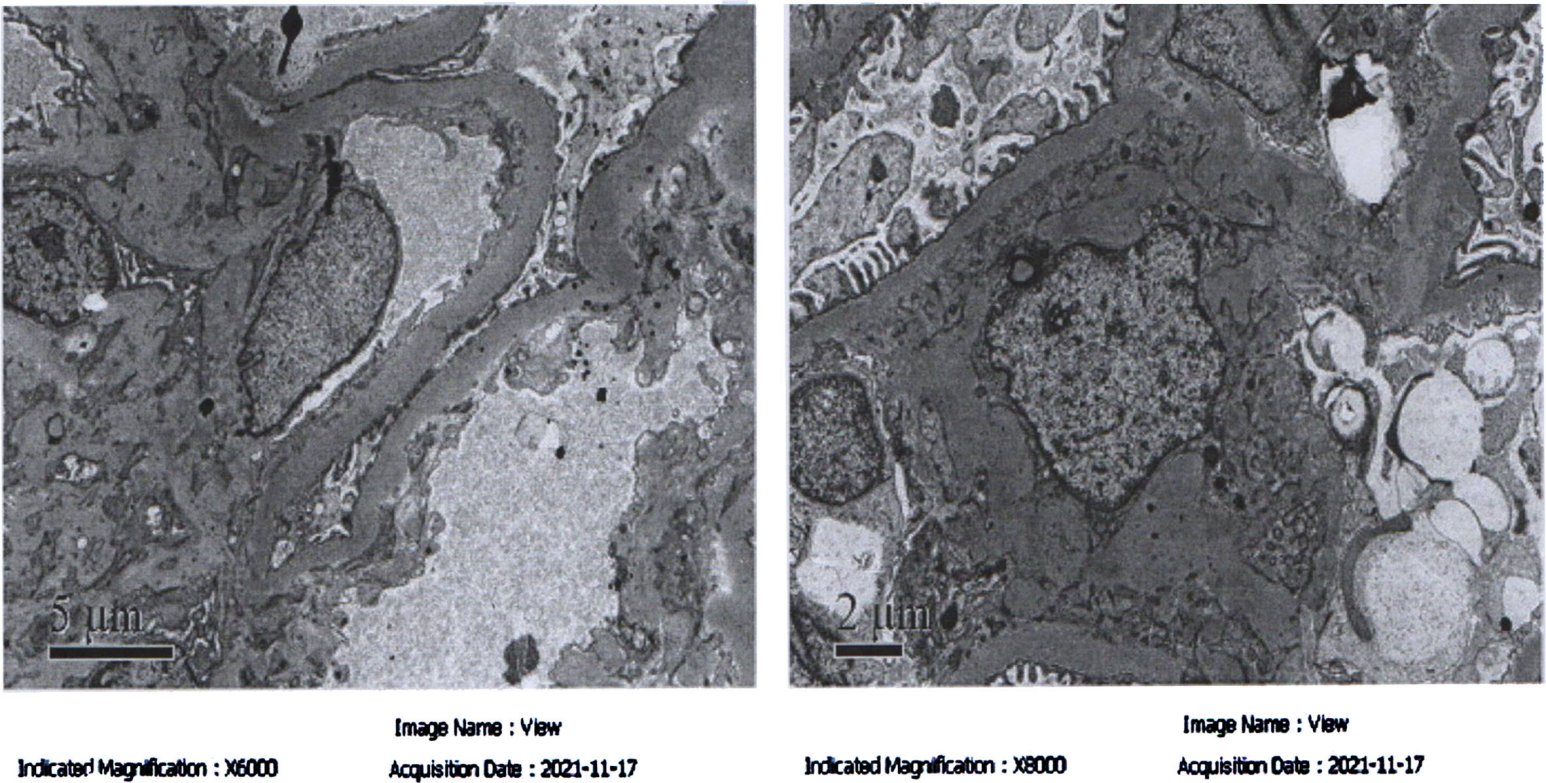
The electron microscope results of the patient. The black arrow indicates the lesions.

Electron microscopy examination showed mild to moderate proliferation of glomerular mesangial cells, mostly homogeneous thickening of the glomerular basement membrane (GBM), occasional small deposits of electron-dense material in the mesangial region, and segmental fusion of epithelial foot processes. The renal tubular epithelial cells exhibited vacuolar degeneration with increased lysosomes and partial atrophy. The renal interstitium was infiltrated by a small number of lymphoid mononuclear cells with collagen fiber proliferation. These results were consistent with mild to moderate mesangial proliferative IgAN (Type IIa), as shown in **Figure 2**. Hormones (methylprednisolone: 32 mg, q.d.) and prophylactic (isoniazide: 0.3 g, q.d.) anti-tuberculosis therapy were conducted for three months, after which NS was found to be partially relieved. The patient is currently undergoing treatment.

## Discussion

This section will address the following suspected diagnoses: 1. DKD; 2. DKD combined with primary glomerular disease; 3. primary glomerular disease.

DKD refers to chronic kidney disease (CKD) caused by DM and is usually clinically diagnosed based on elevated urinary ALB and/or decreased estimated glomerular filtration rate (eGFR) while excluding other CKDs. DKD is clinically characterized by progressive proteinuria and progressive decline in renal function (4). The results of electron microscopy showed homogeneous thickening of GBM and increased mesangial matrix, with no electron-dense deposits, and fusion of podocyte foot processes. As DKD is mainly diagnosed based on clinical criteria, and most patients do not receive a formal evaluation by renal biopsy, misdiagnosis and underdiagnosis are common (5). Clinicians should combine multiple auxiliary examinations to make a comprehensive judgment. In the present study, if considering only the 26-year history of DM with poor BG control and one year of proteinuria, it would be very easy to misdiagnose DKD in the event that the progression of proteinuria was ignored. It was found that the incidence of combined NDKD in the pathology of renal biopsies of patients with DKD could be 12–39%, with MN being the most common, followed by minimal change disease (MCD), acute infectious nephritis, and membranous proliferative nephritis. Patients with DKD combined with IgAN accounted for approximately 34.0–66.9% of DKD combined with primary kidney disease (6).

Pathology should be required under the following conditions to identify the presence of DKD or DKD in combination with primary glomerular disease: 1. patients with a DM duration of less than five years accompanied by urine protein of >1g/24h; 2. patients with microscopic hematuria (>++) or rapid decline in renal function; 3. patients with renal involvement without DR or hematuria of unknown cause; 4. patients with a DM duration of ≥ 5 years and with the recent occurrence of massive proteinuria, but with normal BP.

Renal biopsy revealed that the incidence of NDKD in patients with T2DM ranged from 14% to 82.9%, with an average prevalence of approximately 30% (7). A multicenter study showed that among patients with T2DM who underwent renal biopsy only with high clinical suspicion, the proportion of NDKD alone or DKD combined with NDKD was 33% and 38%, respectively (8). In patients with T2DM, it is important to differentiate and correctly diagnose DKD, NDKD, and DKD combined with NDKD. Another study showed that although DR and a longer duration of DM are important predictors of DKD, NDKD cannot be excluded in these patients. NDKD was diagnosed in 40% of patients with T2DM even in the presence of DR (9); therefore, the course of DM and the existence of DR might not be reliable in the differential diagnosis of DKD from NDKD (10).

IgAN is the most common pathological type in renal biopsy in adults. Primary IgAN is most common in diabetic patients with NDKD (11). IgAN is insidious and mainly affects young adults, but also may occur in children and the elderly. The characteristic immunopathology of IgAN is as follows: IgA-dominant immunoglobulins are diffusely deposited in the mesangial region of the glomerulus in granular or clump-like forms, often accompanied by C3 deposition. Patients with IgAN and DM have different clinical and pathological features, as well as prognoses, compared to those with IgAN alone (12). The present patient experienced partial alleviation of NS after treatment targeted IgAN. It was reported that steroid hormone and immunosuppressive therapy might improve prognosis in at least 50% of patients with NDKD, and that an accurate diagnosis based on pathology could improve or reverse the renal lesion (13, 14). The results of the present study suggest that clinicians should be open-minded and carefully identify patients with long DM duration combined with kidney disease in order to reduce misdiagnosis and missed diagnoses. In the future, several biomarkers, including miR-95-3p, miR-185-5p, miR-1246, and miR-631, are expected to serve as simple and non-invasive tools for use in differentiating DM, DKD, DM combined with IgAN, and DM combined with MN (15).

## Conclusion

Renal biopsy may aid the definitive diagnosis of DKD, NDKD, and NDKD combined with DKD. Precise therapy based on renal pathology might help to improve outcomes in the kidney.

## Data availability statement

The raw data supporting the conclusions of this article will be made available by the authors, without undue reservation.

## Ethics statement

This study was conducted with approval from the Ethics Committee of the Affiliated Hospital of Hebei University. The patients/participants provided their written informed consent to participate in this study.

## Author contributions

Conception and design of the research: Y-LG, YX. Acquisition of data: YX, Y-PZ. Analysis and interpretation of the data: Y-PZ, YH. Statistical analysis: J-DL. Obtaining financing: HC. Writing of the manuscript: Y-LG, YX. Critical revision of the manuscript for intellectual content: HC. All authors contributed to the article and approved the submitted version.
